# On the Diseases of Infants and Children

**Published:** 1850-01

**Authors:** 


### BIBLIOGRAPHICAL NOTICES.

*On the Diseases of Infants and Children.* By Fleetwood Churchill, M. D., M. R. I. A., Hon. Fellow of the College of Physicians, Ireland; Hon. Member of the Philadelphia Medical Society, &c., &c. Philadelphia: Lea & Blanchard, 1850. pp. 613. 1 vol. 8vo.

*A Practical Treatise on the Diseases of Children.* By Francis Condie, M. D., Secretary of the College of Physicians, Member of the American Medical Association, &c., &c. Third edition, revised and augmented. Philadelphia: Lea & Blanchard, 1850.

It is but two months since we welcomed the appearance of Dr. West's valuable work on the diseases of children, and we have now to proclaim the publication of another work on the same subject, by Dr. Fleetwood Churchill, of Dublin, the well known writer on the Theory and Practice of Midwifery and the Diseases of Females. We are unfeignedly pleased to find from the fact that two foreign works of the size and cost of the above, have been issued by the same publishing house within such short space of each other, that the profession in this country is becoming fully aware of the importance of the diseases of children as subjects of distinct and careful investigation.

It appears from the preface of the work we are about to notice, that it has been prepared especially with a view to its publication in this country. The author says: "It is with much gratification that I acknowledge this volume to owe its existence to the solicitations of my excellent American publishers." The fact of the publishers having solicited a work on diseases of children, we suppose to be owing to the great success which has attended the republication, in this country, of Dr. Churchill's previous works; one, that upon the Diseases of Females, having reached a fourth edition; and the other, that upon Obstetrics, being now in the third edition; strong proofs, certainly, of the estimation in which the writer is held amongst us.

There is a statement in the preface upon which we have a remark to make. The author, in referring to the secondary diseases, which are so apt to complicate the various maladies of early life, states that this portion of the history of infantile diseases, " has hardly received the attention it deserves." This assertion is perfectly true in regard to the English and American writers on these subjects, but we are surprised that Dr. Churchill does not do more justice in this matter to the French authors upon children's diseases, and especially to MM. Rilliet and Barthez, who have devoted the most minute, careful and systematic attention to this very point (secondary diseases),—a degree of time and attention, indeed, much greater than Dr. Churchill himself.

Dr. Churchill's work is a large and extensive one, consisting of a full sized octavo of 623 pages. We regret that we are totally unable, from want of space, to give a complete and systematic review of the book, an attention which its intrinsic merits and the high reputation of its author certainly make it deserve. But we are compelled to notice it only generally, and this we shall do with as much faithfulness and regard for its deserts as we are capable of, after having carefully examined its contents.

The work is divided into two parts; the first of which is devoted to a consideration of the physical management of children, and the second to the history of the various diseases to which they are subject.

In the first part, we have observations upon the choice of a nurse, upon weaning, artificial feeding, dress, sleep, medicine, and the nursery and nurses. The remarks upon these subjects are, in general, sensible and judicious. We have but one fault to find with them, and that is that they are scarcely copious enough. Indeed, we doubt whether it is possible for a writer, whose professed object is the *diseases* of children, to devote as much space of a single volume to the physical management of the early period of life as the subject really deserves. Nevertheless, what the author has said upon this subject cannot but prove most useful to those who are not provided with the more complete works especially devoted to it. To those who desire the most thorough information upon this matter, we would recommend either Dr. Andrew Combe's able " Treatise on the physiological and moral management of Infancy," edited by Dr. John Bell of this city, or Dr. Bull's excellent little work on the " Maternal Management of Children in Health and Disease," republished last year in this city, by Messrs. Lindsay & Blakiston.

The second part of the work treats, as has already been stated, of the different diseases to which children are subject. The diseases are classified according to the systems of organs which they affect; which is the division now generally adopted, and which is the most useful and convenient. In this part of the work, constituting, of course, by far the greater portion of the whole, the author passes in review, more or less fully, nearly all the diseases to which children are subject, leaving out, in fact, only scrofula and tuberculosis, the diseases of the urinary organs, typhoid fever, and the fevers of malarious origin.

We have already stated that it was impossible for us to examine the work before us in detail. We have neither the time nor the space. In regard to its general character, we may observe that it shows, like Dr. Churchill's other writings, a very great amount of patient industry, a careful study of, and intimate acquaintance with, the works of other authors, and an honest and conscientious appreciation of the labors of those who have preceded him. It is, indeed, mainly a compilation. We find but little, in proportion to the whole amount of matter, that is evidently the product of the author's own observation and experience. But with this we find no fault, for we are not amongst those who value only entirely original contributions to the science. On the contrary, we think that a careful and honest collection of the results obtained by previous observers, is one of the most useful books that can be offered to the medical public. Doubtless a work like that of MM. Rilliet and Barthez, or that of Dr. West of London, is a more important and agreeable production, as adding much of new to the common stock, than one composed chiefly from materials which have already been placed before the profession, and which must therefore be familiar to many. But, a work like the one before us, when well put together, as this is, is highly valuable to many, and especially to those practitioners, forming unhappily the mass of the profession, who read but little, or who have access to no large and well filled library. Those, therefore, who are placed in this situation, and those who are too busy or too indolent to consult original works, will here find condensed into short sentences and few words, the pith and marrow of many a large and extensive dissertation.

We must beg the reader, however, not to take up the impression from us, that Dr. Churchill's work is wholly a compilation. Far from this. The author often refers to what he has himself seen, and judges by his own experience of the propriety or impropriety of different views that may have been advanced. There is one circumstance in regard to the author's manner of stating his own experiences, which has seemed to us much more liable to serious objections, than the want of large personal contributions to the work: we allude to the fact that when he thus refers to his private experience, it is done in that loose and general manner, which was so much employed in medical writings, before the influence of the modern school of careful observation, and the numerical method, had resulted in the introduction of the more precise and accurate modes of statements now very commonly made use of. This circumstance causes us often to regret, in the work we are noticing, the absence of more minute and particular information in regard to the data upon which the author may have formed his opinion. It is pleasant to a reader to have not only the conclusions of his author, but likewise his premises, in order that he may judge for himself as to the correctness of those conclusions.

Dr. Churchill's work is particularly excellent in the historical and descriptive accounts of disease. Under the head of each disorder he gives a sketch of its history, and then tells us with great faithfulness what has been learned in regard to its nature, causes, symptoms and pathological characters, so that we have laid before us a full and accurate picture of the malady in all its various aspects. We can therefore strongly recommend these portions of the work to all who are desirous of becoming acquainted with the existing state of knowledge in regard to the points just referred to. We cannot say that we have been so well pleased with the manner in which the treatment of the different diseases has been discussed. It seems to us that the author has not devoted a sufficient share of his time and attention to this part of his subject. It is not sufficiently elaborated. The remarks seem rather meagre, and have too much the air of a dry catalogue of what other men have thought and done. Here it is, too, that we we feel especially the absence of that accurate and precise method

3*

of statement, of the general want of which in the work we have already spoken.

After this meagre, and we know, very imperfect notice, of Dr. Churchill's work, we shall conclude by saying, that it is one that cannot fail from its copiousness, extensive research, and general accuracy, to exalt still higher the reputation of the author in this country. The American reader will be particularly pleased to find that Dr. Churchill has done full justice throughout his work, to the various American authors on this subject. The names of Dewees, Eberle, Condie, and Stewart, occur on nearly every page, and these authors are constantly referred to by the author in terms of the highest praise, and with the most liberal courtesy.

The second of the works whose titles head this notice is the popular treatise of Dr. Condie, of which a third edition, revised and augmented by a chapter on Epidemic Meningitis, (or cerebro spinal meningitis,) appeared simultaneously with that of Dr. Churchill. Having already, in notices of previous editions, expressed our favorable opinion of the doctrines of this work, we desire only to reiterate it in this.

We regard the treatise of Dr. Condie as one of the very best of its class, and feel a just national pride in the appreciation of its excellence by the medical profession both at home and abroad, as evinced by the rapid sale of the previous editions, and by the confidence with which it is quoted on all hands. We predict for the present edition increasing favor.

